# Comparison of two methods for analysis of gene–environment interactions in longitudinal family data: the Framingham heart study

**DOI:** 10.3389/fgene.2014.00009

**Published:** 2014-01-30

**Authors:** Yun Ju Sung, Jeannette Simino, Rezart Kume, Jacob Basson, Karen Schwander, D. C. Rao

**Affiliations:** Division of Biostatistics, Washington University School of Medicine in St. LouisSt. Louis, MO, USA

**Keywords:** gene–environment interactions, longitudinal family data, Framingham heart study, interactions in family data, HLM, SNP–alcohol interactions

## Abstract

Gene–environment interaction (GEI) analysis can potentially enhance gene discovery for common complex traits. However, genome-wide interaction analysis is computationally intensive. Moreover, analysis of longitudinal data in families is much more challenging due to the two sources of correlations arising from longitudinal measurements and family relationships. GWIS of longitudinal family data can be a computational bottleneck. Therefore, we compared two methods for analysis of longitudinal family data: a methodologically sound but computationally demanding method using the Kronecker model (KRC) and a computationally more forgiving method using the hierarchical linear model (HLM). The KRC model uses a Kronecker product of an unstructured matrix for correlations among repeated measures (longitudinal) and a compound symmetry matrix for correlations within families at a given visit. The HLM uses an autoregressive covariance matrix for correlations among repeated measures and a random intercept for familial correlations. We compared the two methods using the longitudinal Framingham heart study (FHS) SHARe data. Specifically, we evaluated SNP–alcohol (amount of alcohol consumption) interaction effects on high density lipoprotein cholesterol (HDLC). Keeping the prohibitive computational burden of KRC in mind, we limited the analysis to chromosome 16, where preliminary cross-sectional analysis yielded some interesting results. Our first important finding was that the HLM provided very comparable results but was remarkably faster than the KRC, making HLM the method of choice. Our second finding was that longitudinal analysis provided smaller *P*-values, thus leading to more significant results, than cross-sectional analysis. This was particularly pronounced in identifying GEIs. We conclude that longitudinal analysis of GEIs is more powerful and that the HLM method is an optimal method of choice as compared to the computationally (prohibitively) intensive KRC method.

## INTRODUCTION

The advent of genomewide association studies (GWAS) has revolutionized the field by identifying hundreds of common genetic variants associated with many common complex disease traits (). However, an important and sobering observation is that these identified loci have very subtle effects, thus explaining only a small fraction of the heritability of most complex traits (Manolio et al., 2009). It is increasingly recognized that the near-exclusive focus on main effects has become a barrier to the identification of additional genes underlying these disease traits. Greater emphasis is being placed in recent years on gene–environment interaction (GEI) analyses (Aschard et al., 2012). The identification of GEIs is important for many reasons (Le Marchand and Wilkens, 2008; Thomas, 2010). GEIs or more complex pathways involving multiple genes and environments can explain part of the missing heritability (McCarthy and Hirschhorn, 2008; Manolio et al., 2009; Eichler et al., 2010; Visscher et al., 2012). They can further elucidate the biological networks underlying complex disease risk and enable “profiling” of individuals at highest risk for disease.

Longitudinal family studies have desirable properties for identifying GEIs by combining the features of repeated measures and family studies. A conventional longitudinal study involves the repeated evaluation of one or more measurable traits in a series of unrelated individuals. Longitudinal data with interesting applications and examples are extensively described in several books (Hand and Crowder, 1996; Verbeke and Molenberghs, 2000). The repeated measurements help reduce error, increase statistical power, and provide a means to study the pattern and determinants of systematic changes in a phenotype of interest over time. For the analysis of longitudinal data, linear mixed-modeling framework is commonly used either with maximum likelihood estimation (Laird and Ware, 1982) or with generalized estimating equations (GEEs; Zeger et al., 1988; Zeger and Liang, 1992). In contrast, a cross-sectional family study utilizes phenotypic similarities and differences amongst close relatives to disentangle the genetic and environmental contributions to the trait under study (Khoury and James, 1993). A longitudinal family study further increases the power to resolve the genetic and environmental determinants of traits associated with complex diseases and also to study the corresponding determinants of change in such traits over time (Burton et al., 2005).

However, analysis of longitudinal phenotypes in family data is much more challenging due to two sources of correlations: correlations across longitudinal measurements and correlations among related individuals within families. Most approaches for longitudinal family data are computationally demanding when many subjects are involved, particularly when they need to be executed a large number of times as in the analysis of GWAS data (Kerner et al., 2009). Analysis of GEIs in longitudinal family data further increases the computational burden. In this paper, we compared a methodologically sound but computationally demanding method using the Kronecker model (KRC) with an alternative but computationally more forgiving method using the hierarchical linear model (HLM).

## MATERIALS AND METHODS

### STUDY SAMPLE

In this study, we used the Framingham Heart Study (FHS) SNP Health Association Resource (SHARe) data, as obtained through the database of genotypes and phenotypes (dbGaPs). The FHS is the oldest prospective longitudinal cohort study of cardiovascular risk factors in the USA. To identify the factors that contribute to cardiovascular disease, FHS began in 1948 with the recruitment of an original cohort of 5,209 men and women who were 28–62 years of age at entry (Dawber et al., 1951). Clinic examinations took place approximately every 2 years. In 1971, a second generation of study participants, 5,124 children and spouses of children of the original cohort were enrolled (Feinleib et al., 1975). Clinic examinations took place approximately every 4 years. Enrollment of the third generation cohort of 4,095 children of offspring cohort participants began in 2002 (Splansky et al., 2007). This study obtained informed consent from participants and approval from the appropriate institutional review boards.

### GENOTYPE DATA

Genotype data from the FHS SHARe project include approximately 550,000 SNPs that were genotyped using AffymetrixGeneChip® Human Mapping 500 k Array Set and the 50 k Human Gene Focused Panel. Genotyping involved 10,775 samples (some duplicates) from the three generations of participants (including over 900 pedigrees). Genotype calls were made with the BRLMM algorithm. The genotyping data for the 10,043 samples from 9,354 participants that passed the Affymetrix criteria were additionally checked for sex consistency and consistency with family structure, resulting in genotyping data for 9,274 participants in FHS SHARe. More detailed information is available elsewhere (Cupples et al., 2009).

### STATISTICAL ANALYSES

Longitudinal family studies have more complex phenotypic correlation structure than cross-sectional family studies or longitudinal studies of unrelated individuals, as phenotypes are repeatedly measured among related individuals. Repeated measurements for the same individual are temporally correlated. Phenotypes on related individuals at each time are subject to familial correlations due to shared genetic and environmental effects. Furthermore, phenotypes of related individuals at different time points are also correlated due to both factors.

To account for these sources of correlation, we compare two approaches that belong to the mixed modeling framework (Laird and Ware, 1982), one known for its theoretical soundness and the other known for its computational speed. Our first approach models the full variance–covariance of a phenotypic vector as a Kronecker (KRC) product of two variance-covariance matrices: Σ_visit_
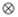
Ω_family_, where Σ_visit_ is the covariance matrix across visits and Ω_family_ is the covariance matrix across family members. Because this methodology has a desirable property that two sources of correlation independently contribute to the overall covariance structure (Galecki, 1994), the KRC is a methodologically sound approach for analysis of longitudinal family data. However, the KRC belongs to the curved exponential family, which makes estimation and testing even more computationally demanding than the unstructured covariance matrix (Roy, 2008). Our second approach is a three-level HLM. Multilevel models are appropriate for data that are organized at more than one level (i.e., nested data). The HLM is also called a mixed model with nested random effects (Berry et al., 2007). We assume that repeated measurements (at the lowest level 1) are nested within individuals (at the next level 2), who are further clustered within families (at the highest level 3). Due to this nested structure, the HLM is computationally more feasible.

For both KRC and HLM approaches in the analysis of longitudinal family data, we use SAS PROC MIXED (Littell, 2006). For a Kronecker product covariance, SAS provides three options: unstructured with compound symmetry (UN@CS), unstructured with order 1 autoregressive (UN@AR(1)), and unstructured with unstructured (UN@UN). In our KRC analysis, we use an unstructured matrix for longitudinal correlation and a compound symmetry matrix for familial correlations. In our HLM approach, we use an autoregressive-moving-average (1,1) covariance matrix for longitudinal correlations and a random intercept for familial correlations(Littell, 2006). When there is no longitudinal data (i.e., a cross-sectional dataset within a single visit), the KRC model with a compound symmetry matrix for familial correlation is mathematically identical to the HLM approach with a random intercept for familial correlations.

To identify GEIs, we consider the following three models for the expected response trait (*Y*):

$$Model\,\;1:E[Y]=\alpha +\beta_{c}C+\beta_{e}E,$$

$$Model\,\;2\,\;:E[Y]=\alpha +\beta_{c}C+\beta_{e}E+\beta_{g}G,and$$

$$Model\,3\,\;:E\left[Y\right]=\alpha +\beta_{c}C+\beta_{e}E+\beta_{g}G+\beta_{ge}GE,$$

where *β*_c_ is the common covariate effect, *β*_e_ is the environmental main effect, *β*_g_ is the genetic main effect, and *β*_ge_ is the GEI effect. For both KRC and HLM approaches described previously, we obtain maximum likelihood (by running SAS version 9.3 PROC MIXED with METHOD = ML) separately using each of the three mean models. Instead of the Satterthwaite and Kenward–Roger degrees of freedom methods, we use EMPICAL option for the estimated variance–covariance matrix of the fixed-effects parameters by using the asymptotically consistent estimator (Huber, 1967; White, 1980; Liang and Zeger, 1986). To test the genetic main effect (*H*_0_: *β*_g_ = 0), we use the likelihood ratio test based on model 1 and 2, which follows a chi-squared distribution with 1 degrees of freedom (df) under the null hypothesis. To test the GEI effect (*H*_0_: *β*_g__e_ = 0), we use the likelihood ratio test based on model 2 and 3 where both models include the genetic main effect. Finally to jointly test genetic main and GEI effects (*H*_0_: *β*_g_ *= β*_ge_ = 0) proposed by Kraft et al. (2007), we use the likelihood ratio test based on model 1 and 3, which follows a chi-squared distribution with 2 df under the null hypothesis. The SAS code is available upon request.

To compare the two methods of analysis and to further investigate whether longitudinal analysis enhances identification of GEIs, we create a dataset that optimize the use of the “original” cohort that was measured 27 times and the “offspring” cohort that was measured seven times (as of when we obtained the FHS SHARe data from dbGaP). We create a dataset that include all seven offspring visits and the corresponding seven visits of the original cohort (matched by the closest visit dates). In addition, for practical reasons related to missing data and time required to complete the analyses, we reduce our dataset to include the most recent five of these seven visits, effectively reducing our dataset to multi-generation families measured at 5-visits. For this investigation, we consider three sets of analyses: 1-visit data (the most recent visit), 3-visit data (by excluding alternate visits), and all 5-visit data. We analyze high density lipoprotein cholesterol (HDLC) concentration in blood serum. We use amount of alcohol (ounces) consumed per week as an interacting covariate. In addition, age, sex, body mass index (BMI), and use of anti-lipid medications are used as common covariates in the analysis of all three datasets described above.

Our analysis is restricted to chromosome 16, where cross-sectional analysis based on a single visit yielded significant results. Out of 15,259 SNPs on chromosome 16, we exclude SNPs that have Hardy–Weinberg equilibrium (HWE) *P*-value less than 10^-^^6^, resulting in 14,026 SNPs. HWE *P*-values are computed based on founders only using PLINK (Purcell et al., 2007), as recommended for family studies. Due to a very long computational time, analysis with KRC for the 5-visit data set is further restricted to a subset of 67 SNPs.

## RESULTS

**Table 1** presents select characteristics of the longitudinal family data that were used for the analysis. Sample size varied across the three datasets used for the analysis: 3,012 subjects for 1-visit, 3,946 unique subjects for 3-visits and 4,190 unique subjects for 5-visits. Our analysis dealt with partially missing longitudinal phenotypes, as indicated by the varying sample sizes. The characteristics were consistent across the three datasets. One notable exception was the use of anti-lipid medication, which was 21% in the 1-visit dataset and about 9% in the 3-visit and 5-visits datasets. Given that the 1-visit data corresponded to the last visit, this is expected because a medication use in longitudinal studies tends to increase over time.

**Table 1:** Descriptive statistics of the longitudinal family data used in the analysis.

Characteristics	1-visit	3-visits	5-visits
Unique individuals, *n*	3,012	3,946	4,190
Observed data, *n*	3,012	9,620	16,480
Male, *n* (%)	1,397 (46.38%)	4,496 (46.74%)	7,703 (46.74%)
Anti-lipids med use, *n* (%)	624 (20.72%)	921 (9.57%)	1,462 (8.87%)
Age, years	60.75 ± 9.25	55.53 ± 11.68	55.89 ± 11.54
BMI, kg/m^2^	28.16 ± 5.31	27.26 ± 5.01	27.28 ± 4.99
Alcohol, oz./week	2.61 ± 3.79	2.81 ± 4.11	2.73 ± 4.06
HDLC, mg/dL	53.74 ± 17.05	51.57 ± 15.75	51.05 ± 15.65

**FIGURE 1:**
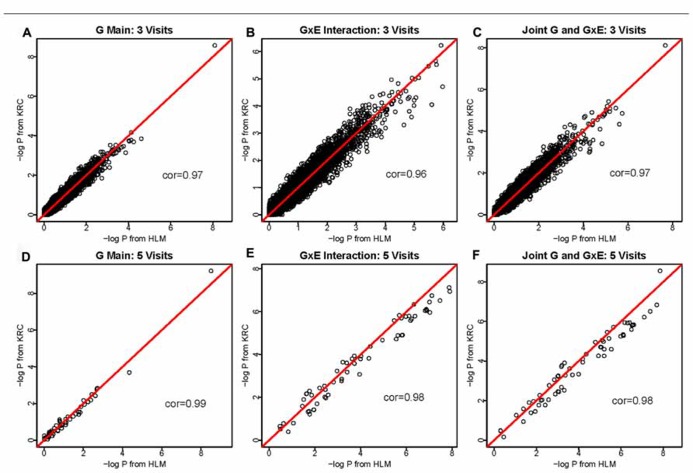
**-log(*P*) values using the HLM (*x*-axis) versus -log(*P*) values using the KRC (*y*-axis) for 3-visit and 5-visit data sets**. Results from both methods were identical for 1-visit data (not shown). The 1st row corresponds to the 3-visit results (**A** for genetic main effects, **B** for GEI effects, and **C** for joint 2 df genetic and GEI effects). Similarly, the 2nd row corresponds to the 5-visits results (**D** for genetic main effects, **E** for GEI effects, and **F** for joint 2 df genetic and GEI effects).

Results were compared between Kronecker (KRC) and HLM approaches for all three hypothesis tests (1 df genetic main effect, 1 df interaction effect in the presence of the genetic main effect, and 2 df joint tests of main and interaction effects) and also for all three data sets. In the 1-visit data, we empirically confirmed that our KRC model provided identical results as our HLM approach for all three hypothesis tests. As shown in **Figure 1**, the results [-log(*P*) values] between KRC and HLM approaches correlated very well for all three tests and for both 3-visit and 5-visit data, providing correlation of over 0.96 in all 6-cases.

Our analysis was run using a cluster of multiple Linux computers. However, to compare CPU time of the two approaches in the three datasets, a subset of analysis with the two approaches was repeated using the identical Linux computer. CPU time with the HLM approach was averaged over 1,500 SNPs for all three datasets. CPU time with the KRC approach was averaged over 1,500, 77, and 6 SNPs for 1-, 3-, and 5-visit data, respectively. As shown in **Table 2**, analysis with the HLM approach was faster than analysis with the KRC approach for all three datasets. This advantage in CPU time was increasingly more pronounced as the data included more visits. Under the identical Linux computer, HLM was 85 times faster for the 3-visit data (1.42 sec vs. 1.43 min per SNP) and 1,000 times faster for the 5-visit data (2.5 sec vs. 42.3 min per SNP).

**Table 2:** CPU time for running analysis with three mean models at each SNP.

Model	1-visit	3-visits	5-visits
Kronecker model	0.55 s	1.43 m	42.33 m
Hierarchical linear model	0.41 s	1.42 s	2.54 s

*Hierarchical linear model runs were averaged over 1,500 SNPs. KRC run times were averaged over 1,500, 77, and 6 SNPs for 1, 3, and 5 visits, respectively.*

**FIGURE 2:**
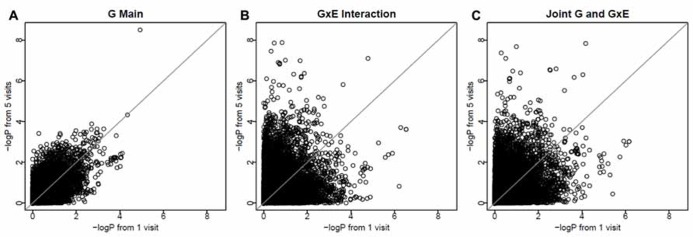
**More visits are better for identifying interactions.** The *x*-axis shows -log(*P*) values using the 1-visit data set, and the *y*-axis shows -log(*P*) values using the 5-visit data set. More SNPs (53, 54, and 55% of SNPs in **A–C**, respectively) are above the diagonal line.

To investigate whether longitudinal analysis enhances statistical significance, we examined the -log(*P*) values using longitudinal data (with 3-visit and 5-visit) and those using one visit only. We found that either longitudinal analysis (3-visit or 5-visit) provided smaller *P*-values, thus leading to more significant results, than the cross-sectional analysis (with 1-visit). In particular, as shown in **Table 3**, the gain in statistical significance was particularly pronounced for identifying GEIs. For example, the number of SNPs with *P*-value <1 × 10^-5^ for identifying GEIs was 8, 10, 31 from 1-, 3-, and 5-visit data, respectively. Similar pattern was also found for jointly identifying the main and interaction effects. **Figure 2** shows results from the 1-visit data set and those from the 5-visit data set. In **Figure 2**, more SNPs are above the diagonal line: 53, 54, and 55% of SNPs had smaller *P*-values from the 5-visit data for identifying main effect, interaction effect, and joint effects, respectively. Similar pattern was also found between results from the 3-visit data and those from the 1-visit data (not shown; available upon request).

**Table 3:** Number of SNPs with *P*-values below thresholds.

	Genetic main effect			Gene–environment interaction effect			Joint main and interaction		
	1-visit	3-visits	5 visits	1-visit	3-visits	5-visits	1-visit	3-visits	5-visits
*P* < 1 × 10^-^^5^	0	1	1	8	10	31	9	10	26
*P* < 1 × 10^-^^6^	0	1	1	4	0	16	3	1	15
*P* < 1 × 10^-^^7^	0	1	1	0	0	6	0	1	4

## DISCUSSION

Complex traits such as blood pressure and cholesterol vary with age and depend on both genetic and environmental factors. For such traits, longitudinal studies are valuable to disentangle genetic and environmental effects. Longitudinal studies also enable the prospective measurement of time-varying factors that are not included in typical genetic studies. The use of longitudinal data in studies of GEIs is particularly appealing because some of the power lost in the analysis of GEIs may be recaptured by the use of longitudinal data. As time-varying covariates are measured with improved precision in longitudinal studies, longitudinal data analysis can enhance the identification of GEIs. With additional features of family studies that utilize phenotypic similarities and differences among relatives to disentangle the genetic and environmental risk factors, longitudinal family studies can further increase the power to resolve the genetic and environmental determinants of traits associated with complex diseases.

However, analysis of longitudinal family data is known to be more challenging due to two sources of correlations arising from repeated measures and family relationships. One may use two-step modeling, in which the repeated measurements are first reduced to a single summary measure per subject by applying some form of data reduction procedure. For example, either quantitative change in a phenotype between the first and last measurements or a slope from a regression model for each individual can be used as a summary measure (Gee et al., 2003). These summary measures may then be analyzed with a standard method for cross-sectional family data. However, such two-step modeling has been shown to be less efficient than single joint modeling that makes use of all the data simultaneously (Gauderman et al., 2003). Moreover, unlike joint modeling, most two-step modeling approaches fail to properly account for uncertainty in the value of the summary measures when fitting the second step. The linear mixed-effect modeling framework that we used in this paper makes use of all the data simultaneously and therefore is preferable (Laird and Ware, 1982).

In this paper, we evaluated two linear mixed-effect models for analysis of longitudinal family data. We compared *P*-values using two models, as they are the most commonly used measure for the association analysis in GWAS setting. Our *P*-values are based on the likelihood ratio tests for the fixed effects that correspond to the genetic main and GEI effects. As suggested by the reviewer, we note that the mixed-effect models are also widely used in quantitative genetics research (Lynch and Walsh, 1998; Mrode and Thompson, 2005). Both heritability estimation (Hopper and Mathews, 1982) and variance component analysis (Amos, 1994) in human genetics research are based on a linear mixed-model that includes a random-effect accounting for unobserved polygenic effects. The restricted maximum likelihood (REML) approach is also commonly used for the estimation of variance components (random effect parameters; Patterson and Thompson, 1971). In addition to SAS, packages such as ASREML (Gilmour et al., 1995) and BLUPf90 (Misztal, 2008) can be used for the REML estimation of the random effect parameters. Also using linear mixed model and the REML approach, the GCTA software has been recently developed to estimate trait variation with GWAS data in order to address the “missing heritability” problem (Yang et al., 2011). A more comprehensive investigation using these packages for the GEI analysis of longitudinal family data would be valuable for improving computational efficiency.

There are several limitations in our work. First, the correlation structure of our Kronecker (KRC) modeling was an unstructured matrix for longitudinal correlation and a compound symmetry matrix for familial correlations (UN@CS). A more appropriate model would be order 1 autoregressive for longitudinal correlation and a compound symmetry for familial correlations [AR(1)@CS]. Because SAS does not provide this option, other packages including those described in the previous paragraph may be more flexible to evaluate various models. Second, we used an autoregressive–moving–average (1,1) covariance matrix for longitudinal correlations and a random intercept for familial correlations in our HLM approach. We chose this because, under no longitudinal data, the KRC model with a compound symmetry matrix for familial correlation is mathematically identical to the HLM approach with a random intercept for familial correlations. Instead of a random intercept, use of kinship matrix may be a better approach to account for familial correlation. Third, our investigation is entirely practically driven. We implicitly assumed that the KRC model is the appropriate model due to its theoretical soundness and evaluated whether the computationally faster HLM approach provides comparable results. Because of this, model selection criteria such as AIC or BIC have not been used to evaluate the differences in model performances. The “sandwich” estimate option that we used for the variance–covariance matrix of the fixed-effects parameters is known to provide robust and asymptotically consistent estimator under miss-specified models (Huber, 1967; White, 1980; Liang and Zeger, 1986). However, as the reviewer noted, the estimates of fixed effects are still a function of the estimated variance components and may change with the random effects specification.

This study provided empirical findings that longitudinal analysis is more powerful and that the HLM is an optimal method of choice as compared to the computationally more intensive Kronecker (KRC) modeling. Our previous work (Shi et al., 2009) presented a proof of concept that the longitudinal approach using HLM can be more powerful than a cross-sectional analysis. The alternative KRC modeling is computationally very intensive. To the best of our knowledge, this is the first paper that provides systematic comparison between HLM and KRC approaches. Our most important finding was that the HLM provided very comparable results but remarkably faster than the KRC, making HLM the method of choice. Our second finding was that longitudinal analysis provided smaller *P*-values, thus leading to more significant results, than the cross-sectional analysis. The gain in statistical significance from longitudinal analysis may be due to the increased number of (repeated) observations. However, the increased sample size alone does not explain why the significance gain was more pronounced for identifying GEIs. GEIs are more difficult to identify and prone to have large standard errors; this is exactly where longitudinal data appear to be more powerful because the repeated measurements help reduce error, thereby increasing statistical power. As our findings were based on an empirical evaluation using data on a single chromosome, they may not be generalized to all situations. As greater emphasis is being placed in recent years on GEI analyses (Aschard et al., 2012), a more comprehensive investigation would strengthen our findings in this timely topic.

## AUTHOR CONTRIBUTIONS

Yun Ju Sung, Jeannette Simino, and D. C. Rao jointly conceived and designed the study. Yun Ju Sung summarized the results and drafted the manuscript. Jeannette Simino led implementation of the methods in SAS and quality control of the results. Rezart Kume performed all analyses and summarized the results. Jacob Basson carried out quality control of the imputations. Karen Schwander prepared all datasets, performed quality control, and quality controlled the results by independently running a subset of the analyses. D. C. Rao provided administrative support and helped Yun Ju Sung in summarizing and interpreting the results. All authors have reviewed and approved the final manuscript.

## Conflict of Interest Statement

The authors declare that the research was conducted in the absence of any commercial or financial relationships that could be construed as a potential conflict of interest.
